# Study of Body Composition and Metabolic Parameters in HIV-1 Male Patients

**DOI:** 10.1155/2014/498497

**Published:** 2014-06-10

**Authors:** Gurudath Gundurao Sreekantamurthy, N Biplab Singh, Th Bhimo Singh, Th Suraj Singh, Karam Romeo Singh

**Affiliations:** Department of General Medicine, Regional Institute of Medical Sciences, Imphal 795004, India

## Abstract

*Background*. HIV patients on highly active antiretroviral therapy (HAART) containing protease inhibitors (PIs) had been often associated with lipodystrophy. However, there are only few studies on association of nucleoside and nonnucleoside reverse transcriptase inhibitors (NRTI and NNRTI) with lipodystrophy. * Study Design*. One hundred and one HIV male patients were categorised into ART naïve (*n* = 22), zidovudine (*n* = 22), stavudine (*n* = 18), tenofovir (*n* = 15), and PIs (*n* = 24) based HAART. Their clinicoepidemiological data had been entered in preformed pro forma. The body composition, using TANITA machine and metabolic parameters like lipid profile, blood sugars was analysed. * Results*. Clinically, lipoatrophy of face was most prevalent in HIV patients on stavudine (15 patients, 83.3%) and PIs (20 patients, 83.3%) based HAART. The mean BMI among study population was in normal range. Excess visceral fat was most prevalent among patients on PIs, 4 patients (16.7%). The waist-hip ratio was significantly higher in PIs (*P* = 0.01) based HAART. There was no significant difference among different study populations in terms of BMI (*P* = 0.917), body water (*P* = 0.318), body fat (*P* = 0.172), bone mass (*P* = 0.200), and muscle mass (*P* = 0.070). Hypertriglyceridiemia was found in stavudine, tenofovir, and protease inhibitors regimens. Low levels of high density lipoprotein (HDL) was found zidovudine, stavudine, and PIs regimens. Fasting and postprandial hyperglycaemia was found PIs and impaired glucose tolerance in stavudine regimen. * Conclusion*. Patients on PIs were associated with truncal obesity and lipoatrophy of face, along with dyslipidemia and hyperglycaemia. Stavudine based regimen is associated with hypertriglyceridiemia and low HDL along with lipoatrophy of face.

## 1. Introduction

Lipodystrophy had been well described and has frequent disturbing side effect with protease inhibitors. The main manifestations of the lipodystrophy (LD) syndrome in patients with normal serum cortisol levels are fat accumulation in the dorsocervical spine (also known as buffalo hump), in the breasts, and in the abdomen or fat reduction in the face and in the limbs. Lipoatrophy may occur isolated or together with fat accumulation. These body changes are accompanied by dyslipidemia and hyperglycemia [1]. However, there are only few reports of hyperlipidemia and lipodystrophy associated with HAART without PI [2, 3]. A recent study by Smith et al. [4] demonstrated an excess of cardiovascular risk factors in HIV patients receiving HAART, and lipodystrophy syndrome has been considered a worrisome factor. This study was performed to analyze the body composition and metabolic variations in HIV-1 infected individuals during treatment with HAART of different ART regimen and without HAART in the north-eastern part of India.

## 2. Subjects and Methods

A hospital based cross-sectional study conducted from October 2011 to September 2013 included random 101 seropositive HIV-1 male patients* aged more than 15 years* who are attending Medicine Out-Patient Department (OPD) and ART centre, Centre of Excellence, Regional Institute of Medical Sciences, for routine check-up on OPD basis. These patients were categorised into the following groups: ART naïve patients (*n* = 22), HIV patients on zidovudine based regimen (AZT and 3TC and NVP or EFV, *n* = 22), stavudine based regimen (d4T and 3TC and NVP or EFV, *n* = 18), tenofovir based regimen (TDF and 3TC and NVP or EFV, *n* = 15), and protease inhibitors based regimen (*atazanavir 300 mg boosted with low dose ritonavir 100 mg*, *n* = 24).* The minimum duration of each ART regimen was for at least 6 months*. The ART was given to patients according to National AIDS Control Organization (NACO) guidelines, India. Patients who were known case of diabetes mellitus, chronic kidney disease, congestive cardiac failure, already on hypolipidemic drugs before starting ART, and suffering from tuberculosis and other active opportunistic infections were excluded from the study.

### 2.1. Data Collection

Detailed history regarding clinicoepidemiological data including, facial dysmorphism (lipoatrophy of face), exposure, HIV infection duration, duration of the present regimen, alcoholism (>80 gms/day), and cigarette smoking (>5 cigarettes/day) were recorded in a preformed pro forma. Anthropometric measurements, like body mass index (BMI) and waist-hip ratio, were measured. Waist circumference was measured at midpoint between lowermost palpable rib and topmost point of iliac crest along midaxillary line, and hip circumference was measured at widest portion of buttocks. Waist-hip ratio >0.9 indicates truncal obesity and high risk for cardiovascular complications [5]. BMI of study population was analysed in accordance with Asian populations [6]. Total body water (in percentage), bone mass (in kgs), visceral fat (in kgs), body fat (in percentage), and muscle mass (in kgs) were analysed using TANITA machine, a body composition guide for inner scan manufactured by Tanita India Private Limited, Mumbai. Visceral fat ranging from 1 to 12 kgs was considered as healthy range and more than 12 as excess [7]. Body fat percentage was analyzed according to weight [8]. Blood biochemistry including lipid profile, kidney function test, and blood sugar levels (random, fasting and postprandial) was performed.

### 2.2. Statistical Analysis Used

SPSS 11 was used to analyse the data. ANOVA and post hoc Dunnet's T3 test were used to determine significant difference among study population.

## 3. Results

### 3.1. Baseline Parameters

*The age range of study population was ART naïve patients, 24 to 51 years; zidovudine based patients, 26 to 53 years; stavudine based patients, 32 to 52 years; tenofovir based patients, 37 to 60  years; and protease inhibitors patients, 32 to 60 years*. The mean duration of HIV infection and the present ART regimen (in months) is shown in Table 1. Lipoatrophy of face was most prevalent in HIV-positive population who are on stavudine based regimen (15 out of 18 patients, 83.3%) and protease inhibitors based regimen (20 out of 24 patients, 83.3%). None of the ART naïve patients had lipoatrophy of face. Three patients (13.6%) on zidovudine based regimen and 3 patients (20.0%) on tenofovir based regimen had lipoatrophy of face (refer Table 2).

### 3.2. Body Composition in Study Population (Refer to Tables 3 and 4)

Mean BMI of different categories of study population were in normal range, and there was no significant difference in mean BMI among different categories of study population (*P* = 0.917). Among study population, excess visceral fat was most prevalent in protease inhibitors group (4 out of 24 patients, 16.7%). The mean total visceral fat among study population was in healthy range, and there was statistically insignificant difference among study population. Though statistically insignificant, mean difference of total visceral fat between PIs (6.21 ± 4.79) and ART naïve (4.09 ± 3.66) patients was about 2.11. Mean body fat percentage was in normal range among study population and did not vary significantly among different study groups (*P* = 0.172). The mean total body water percentage was relatively higher in zidovudine based regimen (61.98 ± 7.86) and stavudine based regimen (61.4 ± 6.63) than other groups (ART naïve regimen was 59.80 ± 9.73, tenofovir based regimen was 58.90 ± 4.9, and protease inhibitors regimen was 57.64 ± 6.99), and there was no significant difference among study population (*P* = 0.318). There was no statistically significant difference among study population in terms of mean bone mass (*P* = 0.200) and mean muscle mass (*P* = 0.070). Waist-hip ratio (mean) in protease inhibitors group was 0.91 ± 0.04, indicating central obesity. The mean waist-hip ratio significantly varied among study population, being higher in HIV patients receiving protease inhibitors (*P* = 0.001).

### 3.3. Metabolic Parameters (Refer to Table 5)

Hypertriglyceridemia was found in stavudine (173.06 ± 48.11), tenofovir (164.07 ± 77.40), and protease inhibitors (249.83 ± 125.3) based regimens. Low HDL was found in zidovudine (32.75 ± 7.19), stavudine (26.72 ± 7.12), and protease inhibitors (32.79 ± 11.0) based regimens. High VLDL was found only in protease inhibitors regimen (33.9 ± 14.10). Patients with protease inhibitors were found to have both fasting (118.04 ± 46.03) and postprandial (205.38 ± 105.26) hyperglycemia. Patients on stavudine based regimen had only postprandial hyperglycemia (145.22 ± 30.11). There was a significant difference in total cholesterol (*P* = 0.001), triglycerides (*P* = 0.000), HDL (*P* = 0.003), LDL (*P* = 0.009), VLDL (*P* = 0.001), RBS (*P* = 0.000), FBS (*P* = 0.001), and PPBS (*P* = 0.000) among study population.

## 4. Discussion

The present study is a cross-sectional comparative data regarding body composition and metabolic parameters derangements among HIV patients receiving protease inhibitors and NRTIs. 83.3% patients receiving protease inhibitors had facial dysmorphism in form of lipoatrophy and 16.7% patients had excess visceral fat with normal total body fat percentage. In addition to this, there was increased waist-hip ratio, more than 0.90, indicating truncal obesity. There were also metabolic abnormalities in the form of hypertriglyceridemia, low HDL, high VLDL, and fasting and postprandial hyperglycemia. These features indicate regional fat redistribution from periphery to visceral resulting in insulin resistance rather than mere fat loss or fat gain. Similar finding was also described by previous study by Dinges et al. [9] and Carr et al. [10]. Mulligan et al. [11] analysed the paired data in HIV infected patients before and after initiating PIs (*n* = 20). They found increase lean body mass and fat mass in protease inhibitor group. They also found significant increase in glucose (+9 ± 3 mg/dL, *P* = 0.0136), insulin (+12 ± 4.9 mg/dL, *P* = 0.023), triglycerides (+53 ± 17 mg/dL, *P* = 0.0069), and total and LDL cholesterol (+32 ± 11 mg/dL and +18 ± 5 mg/dL, *P* = 0.0082 and 0.0026, resp.). In present study, there was no significant difference between the study groups in terms of BMI, total body water, bone mass and muscle mass, and visceral and body fat. Samaras et al. [12] in their international cross-sectional data involving 788 cohorts of HIV patients on HAART noticed 14% prevalence of metabolic syndrome according to International Diabetic Federation criteria and 18% prevalence of the same, according to US national cholesterol education program adult treatment panel III (ATP III) criteria, in patients receiving PIs, the highest being in indinavir and the least in amprenavir. 49% of patients had at least 2 features of metabolic syndrome but did not meet the diagnostic criteria. In present study also, it is emphasising the same fact of insulin resistance in the form of truncal adiposity and hyperglycemia in patients receiving PIs. Mulligan et al. [11] studied 148 HIV-1 patients (116 patients were on protease inhibitors and 32 had never taken protease inhibitors) and in 47 healthy men found that development of syndromic features like central adiposity along with hypertriglyceridemia, hypercholesterolemia, and insulin resistance with type 2 diabetes mellitus occurred mainly with the use of all potent protease inhibitors and hence concluded it to be a class effect.

Current data suggests that abnormal fat distribution occurs in HIV patients receiving non-PIs based regimen. Peripheral fat wasting was noticed in clinically stable asymptomatic HIV patients and may be due to long term use of NRTIs especially stavudine. Lipoatrophy of face (buccal pad fat atrophy) was observed in 83.3% of patients receiving stavudine based HAART, 13.6% of patients in zidovudine group, 20.0% of patients on tenofovir group, and none in ART naïve group. Along with this, stavudine subgroup also had hypertriglyceridemia, low HDL, and postprandial hyperglycemia, which were almost absent in other NRTIs groups. On the contrary, hypertriglyceridemia was also found in the tenofovir group. This may be due to their previous treatment with stavudine based therapy. Saint-Marc et al. [13] in their cross-sectional data on 43 HIV patients on NRTI found peripheral lipodystrophy (Bichat atrophy) in 17 patients (63%) receiving stavudine, 3 patients (18.75%) receiving zidovudine and none in ART naïve. Along with lipodystrophy, patients on stavudine had hypertriglyceridiemia which is similar to result of the present study. Domingo et al. [14], in his study involving 150 HIV patients receiving HAART, 75 stavudine based patients, and 75 zidovudine based patients noticed significant lower body fat in patients taking stavudine. Ninety-four patients had regional fat redistribution in terms of lipoatrophy. Mallal et al. [15] analysed regional fat measured by dual energy X-ray absorptiometry (DEXA) in 161 HIV patients and found progressive subcutaneous fat wasting occurring in stavudine-containing regimens compared with zidovudine-containing regimens (relative risk was 1.085 per month; *P* < 0.0001) and concluded that NRTIs especially stavudine do have an independent contribution to fat wasting. Other body composition parameters like total body water, muscle mass, and bone mass are not significantly affected among study population. HDL was significantly low in ART naïve and all NRTI groups except for tenofovir group. Though LDL was in normal range, it differed significantly (*P* = 0.009) among study population, being high in stavudine based regimen (94.11 ± 12.4) and protease inhibitors (98.3 ± 27.8).

## Figures and Tables

**Table 1:** Duration of HIV infection and duration of ART in different study population.

Parameters	ART naïve	Zidovudine based regimen	Stavudine based regimen	Tenofovir based regimen	Protease inhibitors regimen
Duration of ART in months (mean)	0	45	64	51	111
Duration of HIV in months (mean)	5.1	51.7	75.6	66.3	126

**Table 2:** Prevalence of lipoatrophy of face in different study population.

Lipoatrophy of face	ART naïve *n* (%)	Zidovudine based regimen *n* (%)	Stavudine based regimen *n* (%)	Tenofovir based regimen *n* (%)	Protease inhibitors regimen *n* (%)
Present	0 (0%)	3 (13.6%)	15 (83.3%)	3 (20.0%)	20 (83.3%)
Absent	22 (100%)	19 (86.4%)	3 (16.7%)	12 (80%)	4 (16.7%)

Total	22 (100%)	22 (100%)	18 (100%)	15 (100%)	24 (100%)

**Table 3:** Prevalence of excess and healthy visceral fat among study population.

Visceral fat	ART naive	Zidovudine based regimen	Stavudine based regimen	Tenofovir based regimen	PIs based regimen	Total
Healthy	21 (13.1%)	20 (12.5%)	18 (11.2%)	14 (8.8%)	20 (12.5%)	160 (100%)
Excess	1 (4.2%)	2 (8.3%)	0 (0%)	1 (4.2%)	**4 (16.7%)**	24 (100%)

Total	22 (12.0%)	22 (12.0%)	18 (9.8%)	15 (8.2%)	24 (13.0%)	184 (100%)

**Table 4:** Body composition parameters in study population.

Parameters (mean ± SD)	ART naive	Zidovudine based regimen	Stavudine based regimen	Tenofovir based regimen	Protease inhibitors	*P* value
BMI	20.85 ± 2.35	21.15 ± 2.39	21.48 ± 2.28	20.63 ± 3.39	21.22 ± 3.22	*P = *0.917
Total visceral fat (in Kgs)	4.09 ± 3.66	5.18 ± 4.46	5.11 ± 3.92	6.20 ± 4.16	6.21 ± 4.79	*P = *0.467
Total body fat (in %)	13.60 ± 4.71	12.25 ± 5.81	13.82 ± 5.64	14.96 ± 5.96	16.63 ± 7.71	*P = *0.172
Total body water (in %)	59.80 ± 9.73	61.98 ± 7.86	61.4 ± 6.63	58.90 ± 4.91	57.64 ± 7.69	*P = *0.318
Total muscle mass (in Kgs)	45.05 ± 4.10	47.69 ± 3.53	47.5 ± 5.14	44.46 ± 6.63	44.56 ± 5.05	*P = *0.070
Total bone mass (in Kgs)	2.52 ± 0.23	2.69 ± 0.34	2.73 ± 0.83	2.45 ± 0.32	2.50 ± 0.24	*P = *0.200
Waist-hip ratio	0.87 ± 0.02	0.85 ± 0.04	0.87 ± 0.03	0.86 ± 0.04	0.91 ± 0.04	*P = *0.001

**Table 5:** Metabolic parameters in study population.

Metabolic parameters	ART naïve	Zidovudine based regimen	Stavudine based regimen	Tenofovir based regimen	Protease inhibitors based regimen	*P* value
Total cholesterol	120 ± 34.6	134.77 ± 31.29	154.50 ± 41.4	147.87 ± 37.9	173.46 ± 52	0.001
Triglycerides	117.81 ± 44.1	117.32 ± 38.3	173.06 ± 48.1	164.07 ± 77.4	249.83 ± 125.3	0.000
HDL	38.85 ± 9.1	32.75 ± 7.1	26.72 ± 7.12	37.47 ± 14.6	32.79 ± 11.0	0.003
LDL	76.8 ± 21.8	83.85 ± 14.5	94.11 ± 12.4	87.7 ± 20.8	98.3 ± 27.8	0.009
VLDL	20.86 ± 11.9	21.57 ± 6.2	25.88 ± 11.4	25.53 ± 6.1	33.9 ± 14.1	0.001
RBS	104.05 ± 22.3	97.74 ± 16.2	123.67 ± 35.9	117.53 ± 43.5	188.13 ± 81.8	0.000
FBS	88.86 ± 8.0	82.64 ± 7.6	96.67 ± 13.0	95.60 ± 39.0	118.04 ± 46.0	0.001
PPBS	129.19 ± 26.7	116.86 ± 21.4	145.22 ± 30.1	133.90 ± 57.6	205.38 ± 105.2	0.000

## References

[1] Carr A, Samaras K, Burton S (1998). A syndrome of peripheral lipodystrophy, hyperlipidaemia and insulin resistance in patients receiving HIV protease inhibitors. *AIDS*.

[2] Grunfeld C Disturbances in lipid metabolism due to HIV infection and its therapy.

[3] Thiébaut R, Dabis F, Malvy D, Jacqmin-Gadda H, Mercié P, Daucourt Valentin V (2000). Serum triglycerides, HIV infection, and highly active antiretroviral therapy, Aquitaine Cohort, France, 1996 to 1998. *Journal of Acquired Immune Deficiency Syndromes*.

[4] Smith CJ, Levy I, Sabin CA, Kaya E, Johnson MA, Lipman MCI (2004). Cardiovascular disease risk factors and antiretroviral therapy in an HIV-positive UK population. *HIV Medicine*.

[5] (2008). *Waist Circumference and Waist-Hip Ratio. A Report of WHO Expert Consultation*.

[6] Kanazawa M, Yoshiike N, Osaka T, Numba Y, Zimmet P, Inoue S (2002). Criteria and classi—cation of obesity in Japan and Asia-Oceania. *Asia Pacific Journal of Clinical Nutrition*.

[7] Wang Z Japanese-American differences in visceral adiposity and a simplified estimation method for visceral adipose tissue.

[8] Gallagher D, Heymsfield SB, Heo M, Jebb SA, Murgatroyd PR, Sakamoto Y (2000). Healthy percentage body fat ranges: an approach for developing guidelines based on body mass index. *American Journal of Clinical Nutrition*.

[9] Dinges WL, Chen D, Snell PG, Weatherall PT, Peterson DM, Garg A (2005). Regional body fat distribution in HIV-infected patients with lipodystrophy. *Journal of Investigative Medicine*.

[10] Carr A, Samaras K, Chisholm DJ, Cooper DA (1998). Abnormal fat distribution and use of protease inhibitors. *The Lancet*.

[11] Mulligan K, Grunfeld C, Tai VW (2000). Hyperlipidemia and insulin resistance are induced by protease inhibitors independent of changes in body composition in patients with HIV infection. *Journal of Acquired Immune Deficiency Syndromes and Human Retrovirology*.

[12] Samaras K, Wand H, Matthew L, Emery S, Cooper D, Carr A (2007). Prevalence of metabolic syndrome in HIV-infected patients receiving highly active antiretroviral therapy using International Diabetes Foundation and Adult Treatment Panel III criteria: associations with insulin resistance, disturbed body fat compartmentalization, elevated C-reactive protein, and hypoadiponectinemia. *Diabetes Care*.

[13] Saint-Marc T, Partisani M, Poizot-Martin I (1999). A syndrome of peripheral fat wasting (lipodystrophy) in patients receiving long-term nucleoside analogue therapy. *AIDS*.

[14] Domingo P, Sambeat MA, Pérez A, Ordoñez J, Rodriguez J, Vázquez O (2003). Fat distribution and metabolic abnormalities in HIV-infected patients on first combination antiretroviral therapy including stavudine or zidovudine: role of physical activity as a protective factor. *Antiviral Therapy*.

[15] Mallal SA, John M, Moore CB, James IR, McKinnon EJ (2000). Contribution of nucleoside analogue reverse transcriptase inhibitors to subcutaneous fat wasting in patients with HIV infection. *AIDS*.

